# Supplementary material for: Clinical Concept Embeddings Learned from Massive Sources of Multimodal Medical Data
Source: Pac Symp Biocomput. Author manuscript; Available in PMC 2020 Jan 1. (PMC6922053)
Supplement: supplemental data [file NIHMS1061160-supplement-supplemental_data.pdf]

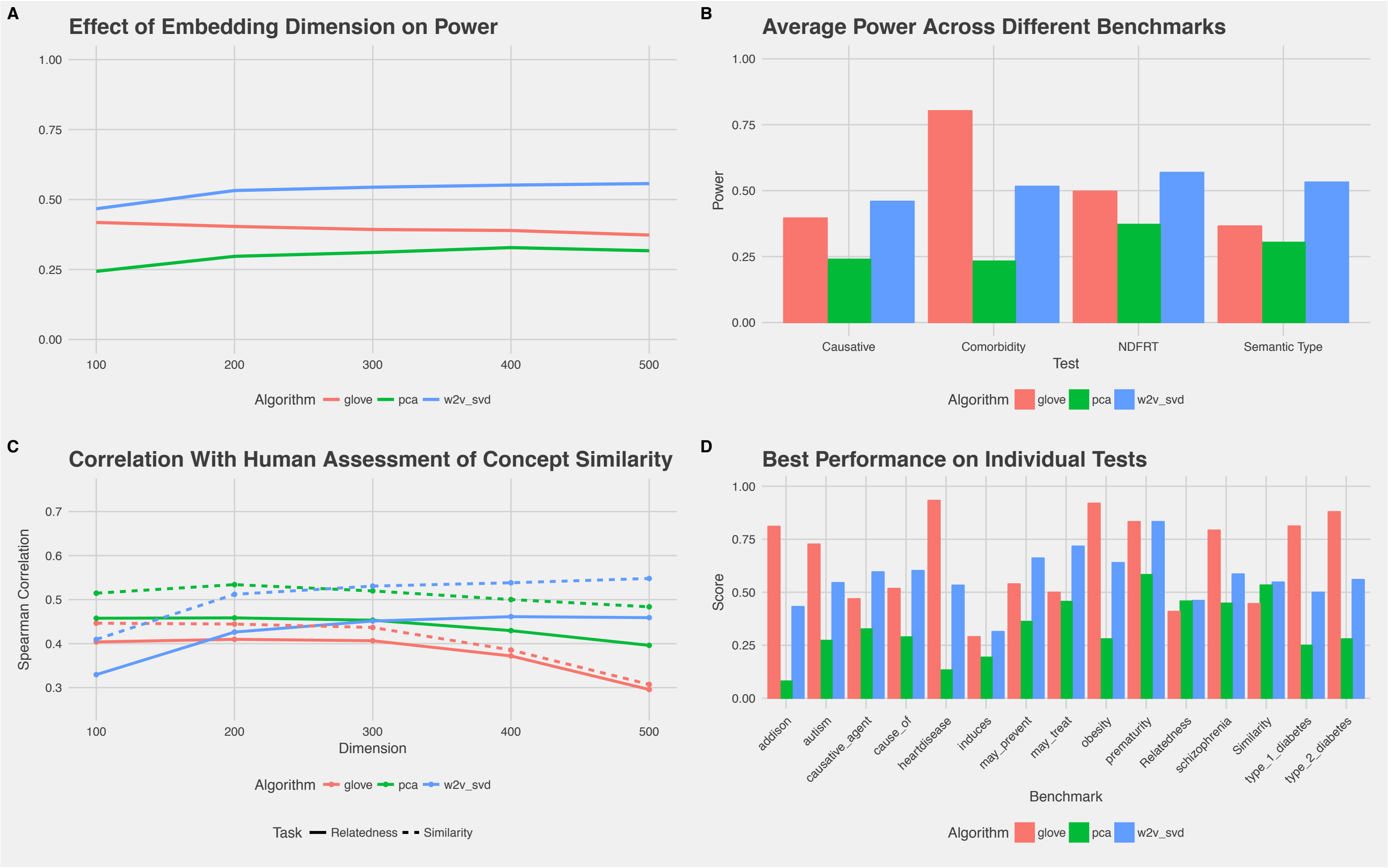

Supplemental Figure 1: Results on combined data. **Panel A**: The effect of embedding dimension on statistical power, averaged across benchmarks. **Panel B**: Power for the best configuration for each algorithm across each benchmark category (standard errors show as black bars). **Panel C**: The effect of embedding dimension on spearman correlation between concept-concept cosine similarity with human assessments of similarity and relatedness. **Panel D**: Best performance by each algorithm on individual tests.

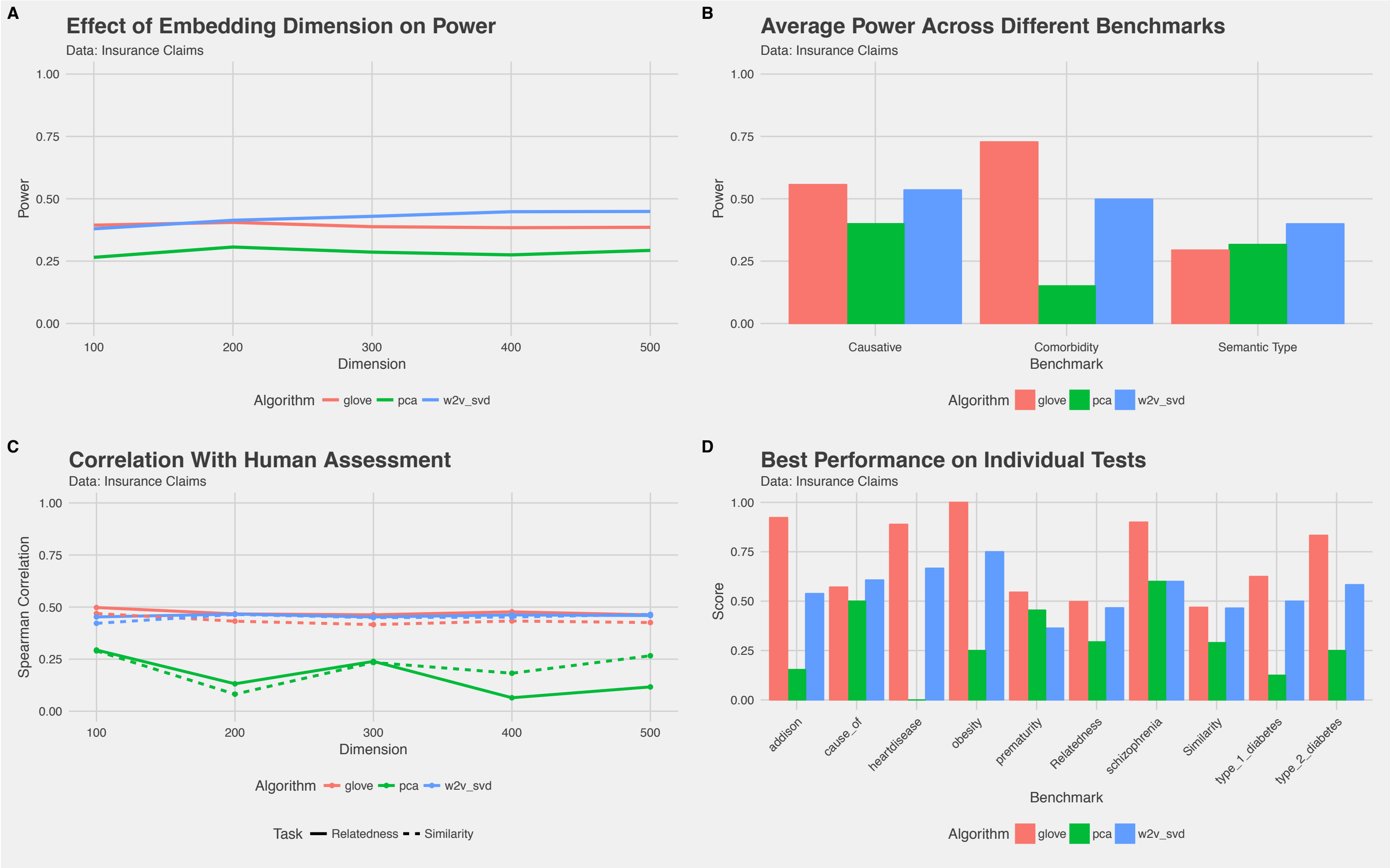

Supplemental Figure 2: Results for GloVe, PCA, and word2vec on the insurance claims data.

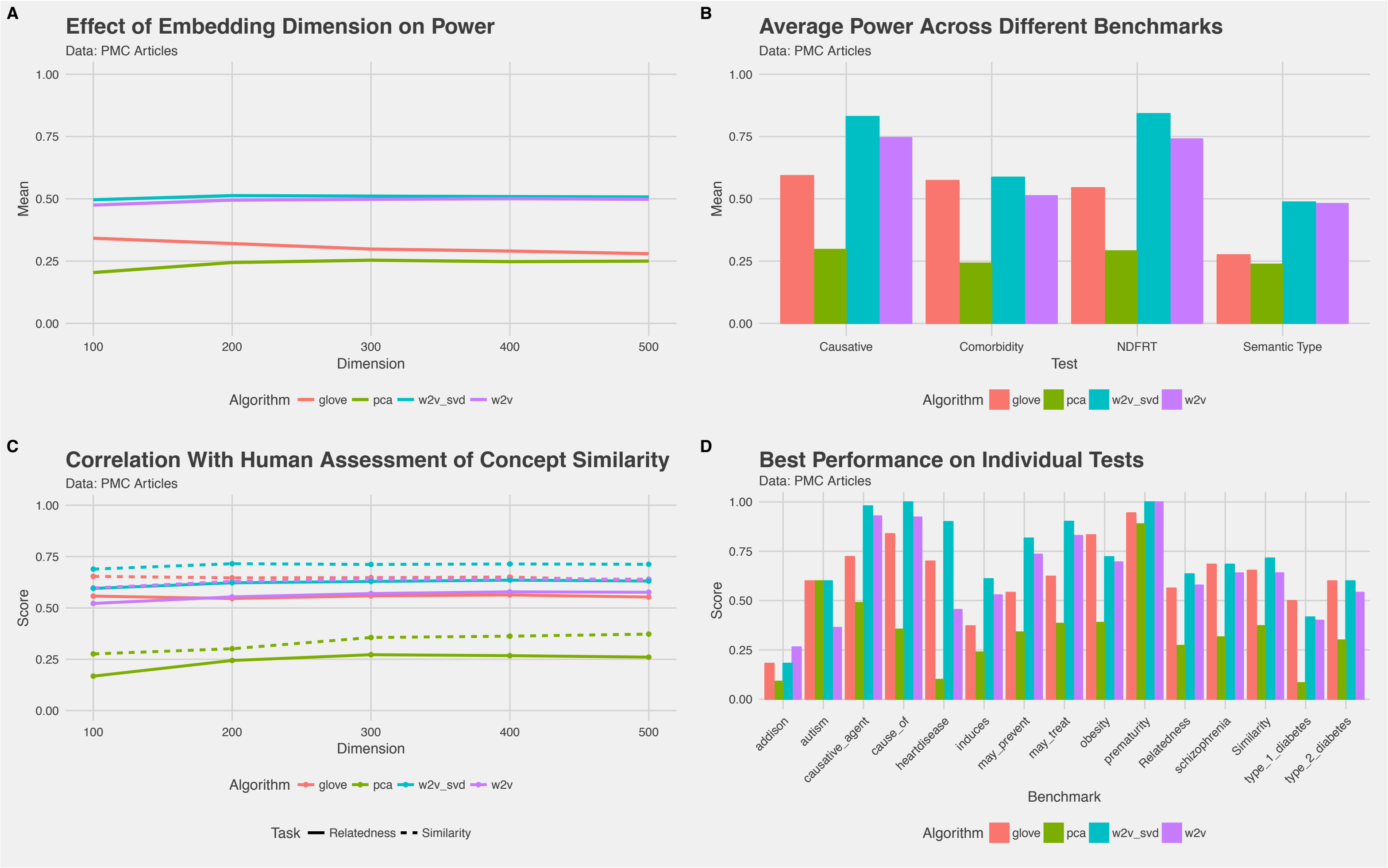

Supplemental Figure 3: Results for GloVe, PCA, and word2vec (SVD and original as implemented in the genism package) on the full text articles from PubMed.

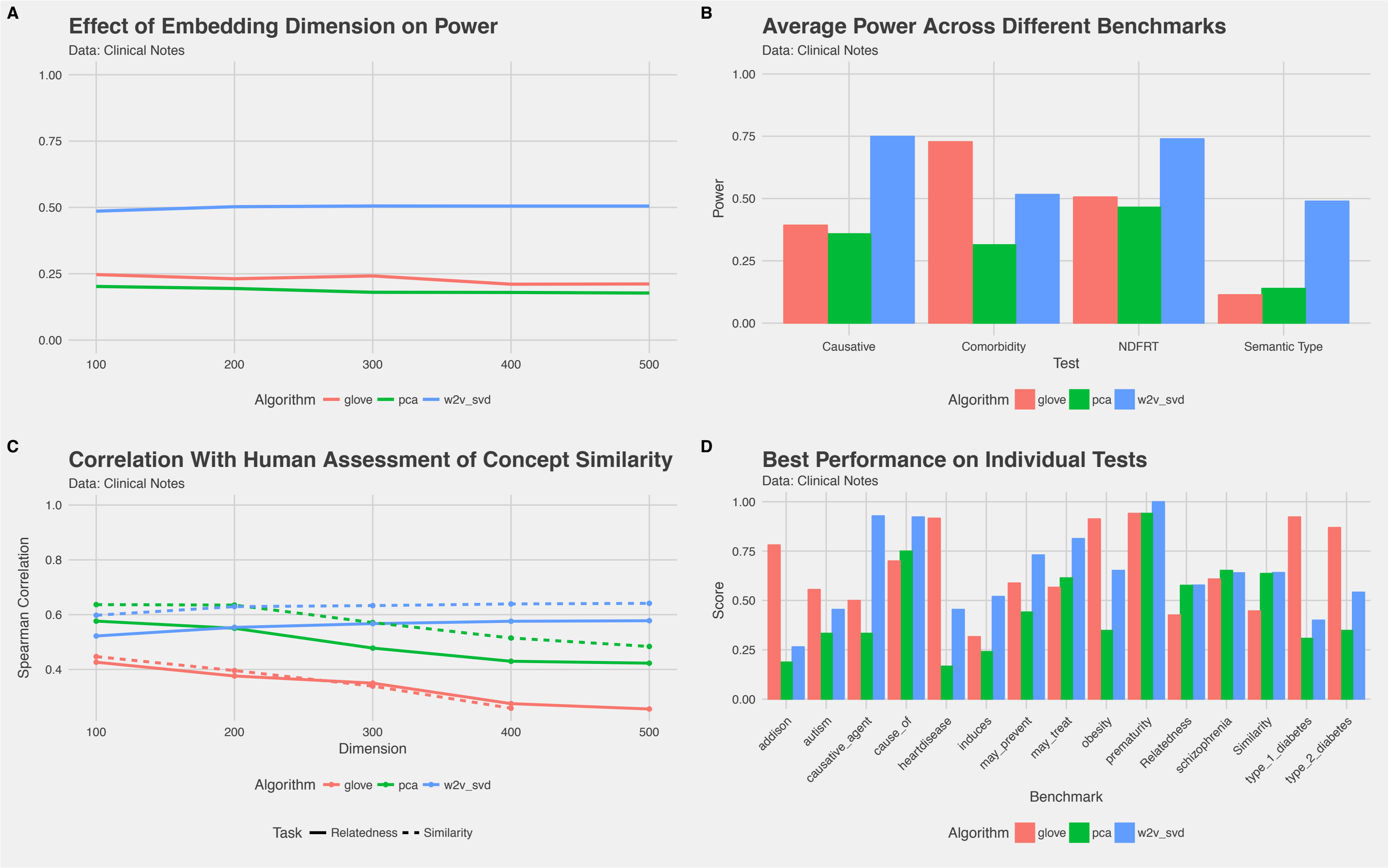

Supplemental Figure 4: Results for GloVe, PCA, and word2vec on the Stanford clinical notes data.
